# Exploring the Science of Shape: How Physical Activity, Sleep, and Stress Affect Body Composition

**DOI:** 10.3390/healthcare13080949

**Published:** 2025-04-20

**Authors:** Krystian Maruszczak, Wojciech Kasperek, Konrad Kustra, Joanna Baran, Maciej Kochman

**Affiliations:** Institute of Physiotherapy, Faculty of Health Sciences and Psychology, Collegium Medicum, University of Rzeszów, 35-215 Rzeszów, Poland; wkasperek@ur.edu.pl (W.K.); kkustra@ur.edu.pl (K.K.); jbaran@ur.edu.pl (J.B.);

**Keywords:** body composition, physical activity, sleep, stress, obesity

## Abstract

In the contemporary context of health challenges, the focus on physical health has become a social and individual priority. Within this framework, body composition emerges as one of the key determinants of physical health, with deviations from normal body composition being associated with numerous pathological conditions that can lead to serious health issues. Consequently, there is an urgent need to synthesize the available knowledge and increase awareness regarding healthy body composition and the factors that shape its components. This narrative review aims to summarize the knowledge regarding the main components of body composition and the key factors that influence their development. The fundamental morphological characteristics and functions of the primary components of body composition—including adipose tissue, muscle mass, and bone tissue—are addressed. Furthermore, the available methods for assessing body composition are outlined. The role of three key factors that influence body composition is outlined, including, but not limited to, physical activity, sleep quality, and stress levels. Additionally, hormonal fluctuations that determine body composition in relation to the variability of these factors are discussed. The review provides evidence-based information that will be valuable both for disease prevention related to non-communicable diseases and for the promotion of health strategies aimed at long-term physical well-being.

## 1. Introduction

**Figure 1:**
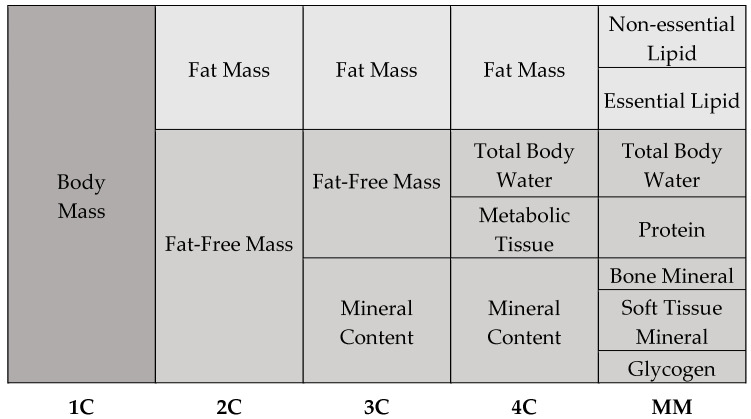
Types of body composition models.

The human body is an extremely complex and yet highly organized structure. Its organization follows a hierarchical structure, starting with the atoms, through the cells, to the tissues and organs. The selected components of the human organism form a heterogeneous body composition. With regard to the chemical composition of the body, five models of body composition are distinguished, including one-compartment, two-compartment, three-compartment, four-compartment, and multi-compartment models (Figure 1) [1,2].

The one-compartment (1C) model provides the foundation for body composition analysis, as it considers body mass as a homogenous whole without discriminating between its many components. According to this idea, the human body is considered as a single mass that includes all tissues and chemicals, regardless of their individual characteristics [1,3]. To create a more accurate picture of body composition, more complex models are used, such as the two-compartment (2C) model. This model assumes that the human body consists of fat-free mass and adipose tissue. Fat-free mass includes water, muscle, bone tissue, and internal organs, while fat mass consists of total and visceral fat. The three-compartment model (3C) extends to the 2C model and is more complex and precise. This model divides body composition into fat mass, fat-free mass, and bone minerals. An even more detailed subdivision than the 3C model is the four-compartment model (4C). This model is very precise and enables detailed differentiation of body composition. It assumes that the human body is made up of fat mass, water, metabolic tissue, and bone minerals. According to current knowledge, the 4C model is considered the “gold standard” of body composition models [1,3]. The last and most complex model of body composition is the multicompartment model (MM). Despite the fact that MM models provide precise body composition measurements, their utilization is constrained by a number of factors. These include the absence of suitable devices, the high costs associated with their use, and the exposure to radiation that they entail [1].

Body composition plays a fundamental role in maintaining health, although its full significance often only becomes apparent in pathological conditions [4]. Each component of body mass has a specific morphological structure and fulfils different functions in the human body. Maintaining appropriate levels of each component and the correct proportions between them is crucial for health. Adipose tissue, for example, fulfils several important functions, including metabolic and endocrinological roles, such as the release of hormones that regulate energy expenditure [5]. However, when excessive amounts of adipose tissue accumulate, as is the case with obesity, it has a detrimental effect on health [6]. Obesity has been linked to numerous health conditions, including neurological deficits [7], insulin resistance, cardiovascular disease [8], and cancer [9], which can contribute to a significant reduction in life expectancy. In addition, reduced muscle mass resulting from an imbalance between catabolic and anabolic processes leads to muscle atrophy [10]. Later in life, this condition can contribute to reduced functional capacity, disability, reduced quality of life, and increased mortality [11,12]. Furthermore, reduced bone mass can lead to osteoporosis, a condition characterized by low bone mineral density and accelerated bone loss [13]. Such a condition is a major risk factor for fractures and falls [14]. Obesity, muscle wasting, and osteoporosis are all components of changes in body composition that lead to serious health consequences and place a significant burden on individual health and healthcare systems [15].

Synthesizing knowledge and increasing awareness within the community regarding body composition is therefore warranted. It is equally important to identify pathways that help maintain body composition within healthy ranges or improve pathological changes in body composition. The present article addresses the topic of body composition, its importance for health, and the essential factors that determine it, including physical activity, sleep, and stress. In-depth analysis is provided of the individual components of body composition, along with an overview of the methods commonly used for their assessment. The review also explores the key morphological characteristics and functions of each component, providing essential insights into their interactions and impact on overall health. The article is also notable for emphasizing not only the physiological aspects of body composition, but also modifiable factors such as physical activity, sleep, and stress that individuals can control and actively influence. While these factors are often discussed individually in existing reviews [16,17], this review goes further by examining how they collectively affect body composition. The manuscript is also enriched by analyzing the hormonal influences on body composition in relation to the variability of these factors, which is an intriguing but often overlooked aspect of the current literature.

## 2. Components of Body Composition

The most important and most abundant component of the human organism is water. The body consists of 50–60% water, with the proportion in men being around 60%, in women 50–55%, and in children 75% of the total body mass. Differences in water content can also be observed between the distinct types of tissue. In muscle tissue, the water content is around 76%, in fat-free mass 73%, and in adipose tissue around 10% [18]. The total water content in the human body is the sum of intracellular and extracellular water and can be expressed as a percentage of body mass or in kilograms. The considerable amount of water in the human body enables it to perform a variety of vital functions. The most important functions include transporting nutrients in the blood and interstitial fluid, maintaining a constant body temperature, lubricating the mouth, joints, and eyes, detoxification, facilitating the function of all organs, and supporting cell metabolism [19]. In addition, water is the main component of cells and is responsible for all processes in the body. Given the numerous functions of water in the human organism, it is crucial to maintain an adequate water balance to ensure an optimal environment for the proper functioning of the body’s organs. Importantly, neglecting the body’s water balance can lead to many negative health consequences, such as fatigue, weakness, dizziness, muscle cramps, rapid breathing, dry skin and mouth, thirst, and palpitations [20].

Alongside water, muscle mass makes up a considerable proportion of body composition. It refers to the total amount of muscle tissue in the body, including skeletal muscle, smooth muscle, and heart muscle. Skeletal muscles play a fundamental role in the human body and account for about 40% of total body mass. They consist mainly of water (75%), protein (20%), and other substances, including minerals, fats, carbohydrates, and inorganic salts (5%) [21]. The content of muscle mass varies according to age and sex. It is assumed that skeletal muscle mass accounts for around 38% of body mass in men and 30% in women [17]. Skeletal muscles fulfil a variety of essential functions in the human body. They are primarily involved in the generation of movement and strength and produce heat, which helps to maintain optimal body temperature. In addition, they play a role in maintaining posture and balance and support the efficient functioning of many processes, including respiration and digestion [22]. Notably, insufficient muscle mass or its loss is associated with slower and poorer wound healing, longer recovery time from illness, lower quality of life, lower basal metabolic rate, and higher healthcare costs [23].

Skeletal muscles are a fundamental component of body composition and fulfil many principal functions in the human body. However, in order for them to perform their basic locomotor function, the presence of bone is required, as bones serve as attachment points for skeletal muscles. Bone tissue makes up about 15% of the total body mass and is the most durable and rigid tissue in the human body. Its structure mainly comprises the cortical bone, which is denser; the trabecular bone, which is more porous; and the bone marrow cavity [24]. Beyond enabling movement, bones provide structural support, protect the internal organs, serve as a reservoir for minerals such as calcium and phosphate, and are responsible for the production of blood cells [24]. Additionally, scientific literature shows that males have larger and stronger bones compared to females [25].

So far, the focus of the discussion on body composition has been on the components of fat-free mass. However, it is important to know that fat mass is a significant part of overall body composition. In the human body, adipose tissue is a complex and heterogeneous structure consisting mainly of fat cells and a small proportion of water [26]. Two main types of adipose tissue are distinguished in the literature: brown and white [27]. Brown adipose tissue is characterized by rich vascularization, and its main function is thermogenesis. White adipose tissue, on the other hand, contains a small number of mitochondria and is found in many areas of the human body. There are two types of white adipose tissue: visceral adipose tissue and subcutaneous adipose tissue. Visceral fat surrounds the internal organs, while subcutaneous fat is found under the skin. In the human body, white adipose tissue functions as an energy reservoir, acts as a heat insulator, and plays an important endocrinological role [27]. This means that fat cells secrete certain hormonal factors in response to physiological signals, which regulate various processes such as appetite and glucose homeostasis. Notably, while a certain amount of adipose tissue is necessary for health, excessive accumulation contributes to the development of various health problems [28].

## 3. Methods of Body Composition Analysis and Assessment

There are currently numerous techniques for analyzing body composition, which can be divided into objective and subjective methods. Objective methods are characterized by high measurement accuracy and use advanced technologies that require special equipment. The most commonly used methods include dual-energy X-ray absorptiometry (DEXA), bioelectrical impedance analysis (BIA), magnetic resonance imaging (MRI), ultrasonography, air displacement plethysmography, and hydrostatic weighing [4,29]. DEXA is considered the gold standard for body composition assessment. This technique is a proven body composition analysis tool that provides precise and standardized measurements of tissue parameters [30]. Due to the excessive costs of DEXA, the BIA method is more commonly used in clinical practice. BIA is a modern, non-invasive, painless, and reliable method of body composition analysis that can serve as a substitute for DEXA [31]. The BIA method works based on the electrical properties of tissues and involves measuring the total electrical resistance of the body, which consists of resistance (passive opposition) and reactance (active opposition) [32]. Notably, tissues in the human body exhibit individual hydration levels and different conductive properties, which translate into varying electrical resistance. Adipose tissue is less hydrated and exhibits no capacitive resistance (reactance), whereas it possesses resistance, or active electrical opposition. In contrast, tissues that are well-hydrated, such as muscle tissue, exhibit reactance. Accurate estimation of total body water content, and consequently assessment of other body composition components, is enabled by understanding these differences in tissue characteristics.

Due to accessibility issues for the above methods, anthropometric measurements are increasingly being used to assess body composition. Anthropometry is a subjective technique for measuring body composition that focuses on the study of the physical dimensions and proportions of the human body. It is a non-invasive method that provides reliable and valuable information about nutritional status and body fat distribution [33]. Commonly used anthropometric measurements include height, body weight, waist circumference (WC), hip circumference (HC), body mass index (BMI), waist-to-hip ratio (WHR), waist-to-height ratio (WHtR), and skinfold thickness measurements. In addition, in response to the growing problem of obesity, derived anthropometric indices have been developed to serve as predictors of fat mass [34]. Among the most widely used are the relative body fat index (RFM), the body roundness index (BRI), the body shape index (ABSI) and the body adiposity index (BAI) [35]. A set of the most common anthropometric indicators along with their characteristics is presented in Table 1.

## 4. Key Determinants of Body Composition

**Figure 2:**
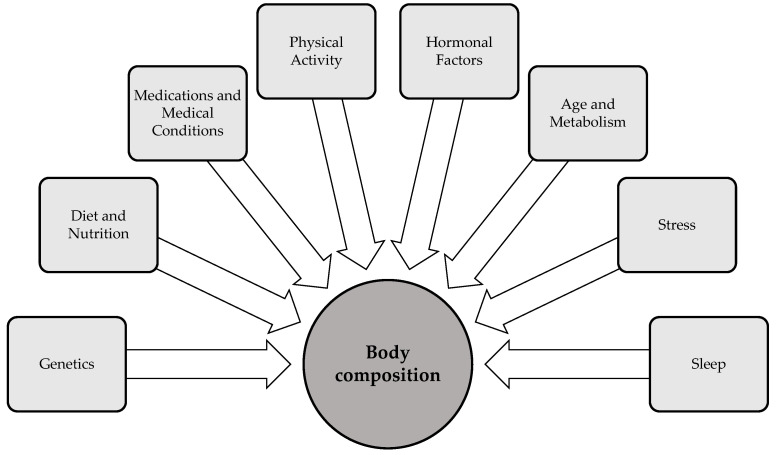
Determinants of body composition [50,51,52].

Body composition is influenced by a variety of factors that interact in complex ways to shape an individual’s health status. Key factors—including genetics, age, gender, physical activity, sleep, diet, and stress—significantly impact body fat distribution, muscle mass, and bone density [50] (Figure 2). Hormonal fluctuations, particularly those involved in metabolic regulation, also play a critical role in shaping body composition by modulating fat storage, muscle development, and energy expenditure. Additionally, environmental factors, such as exposure to endocrine-disrupting chemicals, have the capacity to modulate body composition by altering hormonal pathways and metabolic processes. The following section examines important factors that influence body composition, in particular physical activity, sleep, and stress. While these factors are not the primary determinants of body composition, they are modifiable variables that individuals can actively regulate to influence their body composition.

### 4.1. The Role of Physical Activity in Determining Body Composition

Physical activity plays a key role in building muscle mass, and its effect is well documented in the literature. According to the available scientific data, regular physical activity contributes to an increase in the rate of muscle protein synthesis [53], an improvement in muscle strength and mass [54], and an improvement in cardiovascular and respiratory endurance and functional abilities such as walking speed [55]. Exercise also increases muscle cell sensitivity to insulin, improves glucose tolerance, and supports glycogen storage [56,57]. Moreover, an association has been demonstrated between physical activity and improved mitochondrial function, increased metabolic flexibility, and overall better muscle health. These factors collectively contribute to better metabolic regulation and overall physical health.

In addition to the well-known role that physical activity plays in modulating muscle mass, it is also fundamental in shaping the morphology and function of adipose tissue. Scientific reports indicate that regular physical activity promotes weight loss by reducing adipose tissue and adipocyte size [58]. It also enhances lipolysis and insulin sensitivity within adipose tissue [59], while decreasing lipid storage, lipogenesis, and inflammation. Moreover, consistent physical activity improves the profile of inflammatory markers [60], leading to a reduction in overall body mass and contributing to better metabolic health.

In the context of body composition, physical activity plays a key role not only in the regulation of muscle and adipose tissue content, but also in bone remodeling processes. The scientific literature shows that physical activity improves the mineralization of bones [61] and increases their mechanical strength [62]. This phenomenon results from the fact that the regulation of bone turnover is closely linked to the mechanical stress to which the bones are subjected during physical activity. This mechanical stress is a crucial factor that influences the activity of bone cells, which convert mechanical stimuli into intercellular signals and trigger a cascade of molecular events that lead to increased bone turnover. Regular engagement in physical activity also increases the proliferation of chondrocytes and enhances their differentiation, as well as reduces bone resorption [63]. In the context of bone health, it is noteworthy that physical activity has a positive influence on bone tissue at different ages. In youth, physical activity promotes the growth plates of long bones, while in old age it plays a crucial role in the prevention of osteoporosis [64,65]. Importantly, certain types of physical activity, such as cycling and swimming, do not have a positive impact on bone mineral density. These activities, although beneficial for overall fitness, do not provide the same bone-loading stimulus as weight-bearing exercises, which are essential for maintaining or increasing bone mineral density, particularly in females [66]. In Table 2, the effects of physical activity on the components of body composition are summarized.

### 4.2. The Role of Sleep in Determining Body Composition

The sleep process is thought to play a significant role in the regulation of skeletal muscle mass [67]. This occurs through numerous pathways responsible for muscle protein synthesis and degradation. Remarkably, maintaining a stable level of muscle mass depends on the balance between anabolic and catabolic processes. This means that the equilibrium between protein synthesis and degradation processes will determine the maintenance of muscle mass; a dominance of protein synthesis processes will lead to muscle hypertrophy, whereas a predominance of protein degradation processes will cause muscle wasting. The scientific literature indicates that sleep deprivation is associated with lower muscle mass [68] and muscle strength [69]. Furthermore, shorter sleep duration is associated with greater muscle mass loss during weight loss [70]. The regulation of muscle mass during sleep is largely dependent on the secretion of hormones such as growth hormone, testosterone, and insulin-like growth factor (IGF-1), which have an anabolic effect and significantly influence muscle protein synthesis, ultimately leading to an increase in muscle mass. On the other hand, catabolic substances such as cortisol are released during sleep and contribute to a reduction in muscle mass. The available scientific evidence also suggests that poor sleep quality is associated with a slower decline in cortisol levels in the morning and later in the day, as well as elevated cortisol levels in the evening [71]. This phenomenon is related to the detrimental effect of cortisol on muscle mass due to its high activity in catabolic processes [72].

Beyond its influence on muscle mass, sleep also plays a crucial role in modulating body fat levels. It is generally believed that the development of excessive fat accumulation is primarily due to low levels of physical activity and poor dietary choices. The role of sleep in determining body fat is often underestimated, but numerous studies have confirmed an association between body fat indices and sleep deprivation [73,74]. In fact, sleep deprivation is associated with higher levels of body fat in different age groups [75,76]. It is hypothesized that the mechanism underlying the increased fat mass associated with sleep disturbance involves changes in endocrine function towards a hormonal pattern conducive to fat storage, such as elevated morning cortisol levels. Another mechanism linking sleep and excess fat accumulation is the regulation of food intake, which is modulated by two hormones: leptin and ghrelin [77]. Leptin is a hormone secreted by fat cells that signals the body’s energy status to the brain. In contrast, ghrelin, which is mainly secreted by the stomach, stimulates appetite and leads to increased food intake in individuals. The available scientific literature also suggests that sleep deprivation is associated with increased ghrelin levels [78]. Moreover, in contrast to the anabolic effect of growth hormone on most human tissues, its effect on adipose tissue promotes catabolic processes, thereby increasing lipolysis [79].

**Figure 3:**
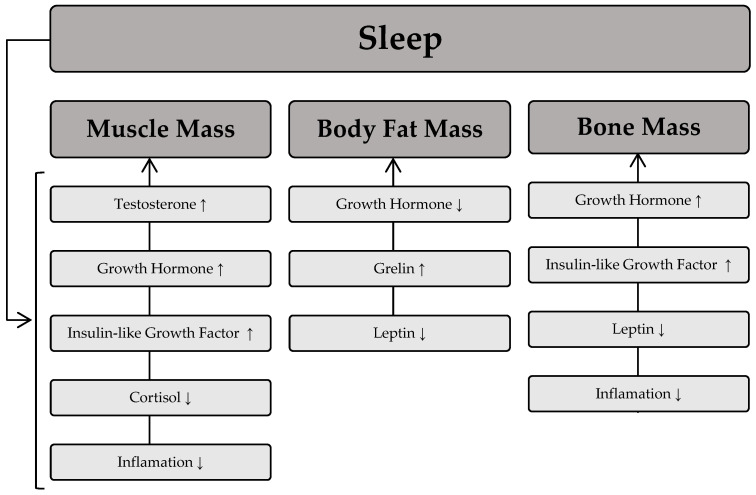
Relationship between sleep and the influence of specific hormones on body composition components. Sleep process → induces fluctuations in specific hormones, which then influence body composition components. An upward arrow (↑) denotes an increase in the respective body composition components as a result of the hormone, while a downward arrow (↓) indicates a decrease in the respective body composition component due to the hormone.

In addition to its effects on muscle mass and adipose tissue, sleep has been shown to be a crucial factor in the regulation of bone turnover. According to recent scientific reports, both long and short sleep duration are associated with low bone mineral density and osteoporosis [80,81]. One of the mechanisms underlying the effect of sleep on bone health is hyperactivity of the sympathetic nervous system. In addition, short sleep duration is associated with an inflammatory state that contributes to low bone mass. Chronic activation of the sympathetic nervous system is also thought to increase levels of leptin in the blood, leading to a reduction in bone mass, particularly cortical bone thickness [82]. On the other hand, good quality sleep promotes increased secretion of growth hormone, which increases the proliferation and differentiation of chondrocytes, thereby benefiting bone health. Figure 3 depicts the relationship between sleep and the influence of specific hormones on body composition components.

### 4.3. The Role of Stress Level in Determining Body Composition

Stress is a physiological and psychological reaction to perceived challenges or threats that disrupt homeostasis [83]. It can be divided into eustress (positive) and distress (negative). Eustress motivates action and mobilizes resources, while distress depletes resources and is detrimental to health [83]. Excessive stress exceeds the body’s ability to adapt and leads to chronic tension, health problems and reduced quality of life. Its characteristic features include a long duration, high intensity, and the lack of effective coping mechanisms that prevent the body from restoring its balance [83].

The literature suggests that prolonged exposure to high levels of stress induces a number of detrimental changes in skeletal muscle morphology and architecture. This occurs because when an individual experiences chronic stress, the hypothalamic–pituitary–adrenal (HPA) axis becomes overactive, leading to excessive cortisol secretion and a systemic inflammatory state. Such conditions promote catabolism muscle protein, [84] induce oxidative damage, reduce ribosome concentration, decrease mitochondrial biogenesis, and impair mitochondrial function [85]. As a result of these changes, a significant decrease in muscle mass and strength is observed, as well as the loss of normal movement patterns [84].

Chronic exposure to high levels of stress not only affects skeletal muscle but also has several adverse effects on adipose tissue. Prolonged stress is responsible for adipocyte hypertrophy and hyperplasia, disruption of adipokine secretion, and fat accumulation, particularly in the abdominal region [86]. Furthermore, long-term exposure to stressors is associated with a chronic inflammatory state characterized by elevated levels of inflammatory cytokines such as tumor necrosis factor-α (TNF-α) and interleukin-6. Scientific reports also suggest that the prolonged hypercortisolemia associated with chronic stress is associated with an increased risk of obesity [87].

**Figure 4:**
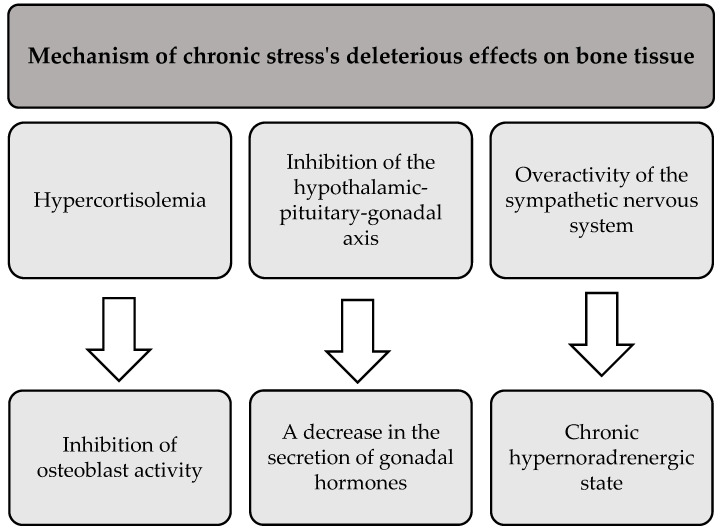
The processes underlying the detrimental effects of chronic stress on bone tissue.

In addition to its negative effects on skeletal muscle and adipose tissue, prolonged stress also adversely affects bone health. The literature suggests that chronic exposure to stress leads to bone demineralization and disruption of bone architecture. This results in reduced bone mineral density and increased susceptibility to fractures and breaks [88]. Three main mechanisms that mediate the deleterious effects of chronic stress on bone tissue are presented in Figure 4 [88,89].

**Figure 5:**
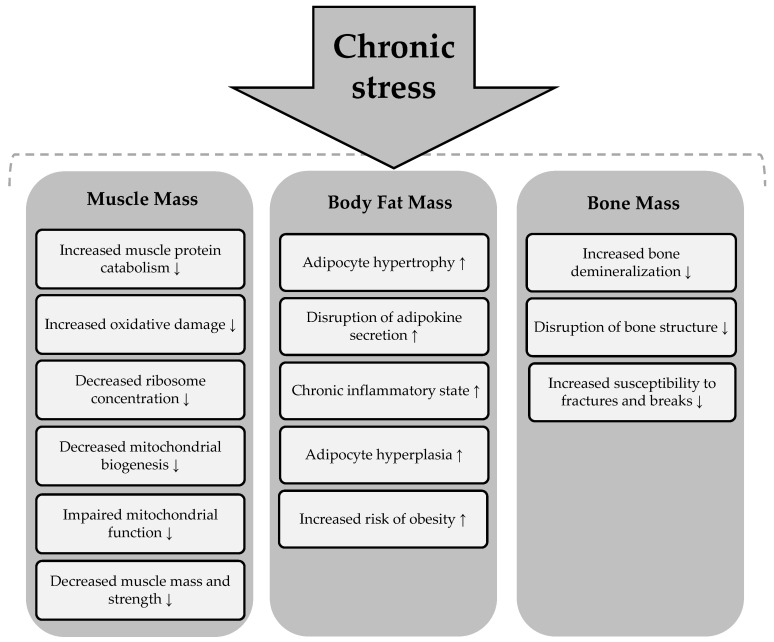
Effects of chronic stress on body composition components. Chronic stress → induces a process or condition that subsequently affects the components of body composition. An upward arrow (↑) indicates an increase in the corresponding body composition component as a result of the process, while a downward arrow (↓) signifies a decrease in the respective body composition component due to the process.

Chronic stress can lead to disturbances in all body composition parameters, including adipose, muscle, and bone tissue. Its detrimental effects are reflected in overall health, contributing to an increased risk of various health conditions [90]. The summary of the effects of chronic stress on body composition parameters is presented in Figure 5.

## 5. The Interplay Between Physical Activity, Sleep, and Stress

The interactions between physical activity, sleep, and stress have attracted increasing scientific interest due to their joint influence on physiological homeostasis and general health. All three factors that contribute to the regulation of body composition exhibit complex mutual interactions.

Physical activity extends beyond its impact on overall health and body composition to encompass a broad range of physiological processes, including the regulation of sleep. There is empirical evidence that regular physical activity contributes to improvements in sleep quality and duration [91,92] and is an effective non-pharmacological intervention for sleep disorders such as insomnia [93]. The underlying mechanisms involve modulation of neuroendocrine pathways, including upregulation of melatonin secretion, attenuation of stress-related physiological responses and improvement of mood. Together, these factors form a positive feedback loop in which improved sleep further facilitates adherence to physical activity and reinforces its beneficial effects [94].

Chronic stress, on the other hand, disrupts this balance by inducing widespread physiological dysregulation, particularly in sleep architecture. There is evidence of a strong inverse relationship between stress intensity and sleep parameters, with increased stress correlating with shortened sleep duration [95], impaired sleep quality [96], and increased incidence of insomnia [97]. The pathophysiological mechanisms underlying this association are multifactorial and include dysregulated activity of the hypothalamic–pituitary–adrenal axis, excessive cortisol secretion, and subsequent suppression of melatonin secretion [98]. Persistently elevated cortisol levels impair melatonin synthesis and disrupt the onset and consolidation of sleep [99]. Stress also increases sympathetic nervous system activity, marked by heightened noradrenaline and adrenaline release, which sustains physiological arousal and further impairs sleep regulation [98]. Together, these neurobiological changes contribute to sleep disorders and reinforce the harmful interplay between stress and sleep homeostasis.

Recent findings suggest that the interaction of physical activity and sleep could have a synergistic effect on body composition. While both factors contribute independently to the regulation of body weight and metabolic health, new studies have begun to investigate how their interaction may improve these outcomes. For instance, a randomized controlled trial by Jåbekk et al. examined the effects of a 10-week resistance training program combined with sleep education [100]. Participants who took part in both interventions showed a significantly greater reduction in fat mass than those who exercised alone, highlighting the added benefit of optimizing sleep alongside physical training. Similarly, Moradell et al. conducted an eight-month multicomponent intervention in older adults combining structured exercise with sleep quality monitoring [101]. The results demonstrated a 10% reduction in body fat percentage and a 47% improvement in sleep quality in the intervention group, suggesting that improvements in sleep may enhance the effects of physical activity on body composition. The results support the hypothesis that combining sleep behavior modification with physical activity may yield more positive changes in body composition than using either factor in isolation.

## 6. Future Directions

The necessity for additional longitudinal studies to enhance comprehension of the long-term effects of physical activity, sleep quality, and stress on body composition is the primary area requiring further research. While extant research has furnished valuable insights into the immediate effects of these factors on body composition, the long-term impacts remain less well understood. Longitudinal studies can assist in identifying patterns of change in body composition over extended periods, thereby revealing how lifestyle factors contribute to the maintenance or deterioration of health across various stages of life. The exploration of potential cumulative effects—such as prolonged periods of physical inactivity, poor sleep, or chronic stress potentially leading to more significant shifts in body composition—is a future avenue for research in this area.

The role of hormonal fluctuations and their complex interactions with lifestyle factors requires further exploration, for example through advanced biomarker profiling and omics technologies. Hormones such as cortisol, insulin, and leptin significantly impact body composition [102]; however, the mechanisms underlying these interactions could be explored in greater depth. The application of advanced omics approaches has the potential to facilitate the identification of biomarkers that reflect these hormonal changes and their effects on fat, muscle, and bone tissue.

Moreover, it is becoming increasingly evident that environmental influences and gut microbiota are significant determinants of body composition [103]. Environmental exposures, such as endocrine disruptors, may alter hormonal regulation, while gut microbiota influences metabolism and fat storage. Investigating these complex interactions in future studies will provide valuable information in the field of body composition determinants. This will facilitate the optimization of body composition and the prevention of metabolic diseases.

Finally, it is also recommended that future research endeavors concentrate on the exploration of the dynamic interplay between body composition parameters, and the way their alterations contribute to the onset and progression of conditions such as cardiovascular disease, diabetes, and metabolic syndrome. Specifically, it would be valuable to investigate the role of sarcopenic obesity, where a loss of muscle mass occurs alongside an increase in fat mass, and how this condition influences metabolic dysfunction. This area represents an interesting direction for future exploration.

## 7. Conclusions

Body composition is inherently influenced by various factors, with physical activity, sleep, and stress being among those most amenable to individual regulation. These factors represent modifiable determinants that individuals can actively manage. Regular physical activity enhances muscle mass and strength, reduces adiposity, and improves bone mineral density and overall skeletal health. Adequate sleep is essential for maintaining optimal body composition, as sleep deprivation disrupts metabolic processes, increases fat accumulation (particularly in the abdominal region) and impairs muscle recovery. Chronic stress induces prolonged elevations in cortisol, contributing to increased visceral fat storage and muscle catabolism, while also influencing eating behavior and promoting weight gain. It is evident that these three modifiable factors play an important role in determining body composition, with modifying them being instrumental in achieving improvements in body composition, consequently enhancing overall health and preventing various diseases, notably non-communicable diseases.

All things considered, addressing these factors is essential, as interventions aimed at increasing physical activity, enhancing sleep quality, and mitigating stress can lead to significant improvements in body composition, thereby contributing to overall health optimization. These interventions also can help prevent or manage chronic conditions, improve mental well-being, and enhance quality of life, making them critical components of a holistic approach to health.

## Figures and Tables

**Table 1:** The most common anthropometric indicators.

Indicator	Formula	Description	References
BMI	BMI = BW/h^2^ where: BW—body weight [kg]; h—height [m]	An index used to classify body weight and assess nutritional status. It is used as a predictor of potential future health problems associated with insulin resistance, hypertension, diabetes, and other pathological conditions.	[36,37]
WHR	WHR = WC/HC where: WC—waist circumference [cm]; HC—hip circumference [cm]	A parameter used to assess body fat distribution and health complications associated with excess body weight, such as cardiovascular disease and diabetes.	[38,39]
WHtR	WHtR = WC/h where: WC—waist circumference [cm]; h—height [cm]	A simple and effective index for identifying health risks, particularly as a screening tool for obesity and cardiometabolic risk.	[40,41]
RFM	RFM = 64 − (20 × h/WC) + (12 × gender) 1—women 0—men Where: h—height [m]; WC—waist circumference [m]	A modern, simple, and practical tool used to estimate the distribution of body fat in the human body.	[42,43]
ABSI	ABSI = WC/(BMI^2/3^ × h^1/2^) where: WC—waist circumference [m]; h—height [m]; BMI [kg/m^2^]	An index used to assess fat distribution, particularly in the abdominal area. It is a predictor of cardiovascular events and allows effective stratification of mortality risk.	[44,45]
BAI	BAI = (HC/h^1.5^) − 18 where: HC—hip circumference [cm]; h—height [m]	An index that estimates body fat percentage. It can be useful in situations where precise measurement of body weight is challenging.	[46,47]
BRI	BRI = 364.2 − 365.5 × (1 − (WC/πh)^2^)^1/2^ Where: WC—waist circumference [cm]; h—height [cm]	An index used to estimate body fat in humans and to determine the percentage of visceral fat. It serves as a prognostic tool for overweight and obesity and facilitates visual comparison of body types.	[48,49]

**Table 2:** Effects of physical activity on body composition components.

Component of Body Composition	Effects of Physical Activity
Muscle Mass	↑ Protein synthesis ↑ Insulin sensitivity ↑ Glucose tolerance ↑ Glycogen storage ↑ Mitochondrial function ↑ Metabolic flexibility ↑ Muscle strength and mass
Fat Mass	↑ Lipolysis ↑ Insulin sensitivity ↓ Adipocyte size ↓ Lipid storage ↓ Lipogenesis ↓ Inflammation ↓ Body mass
Bone Mass	↑ Proliferation of chondrocytes ↑ Differentiation of chondrocytes ↑ Bone mineralization ↑ Mechanical strength of bone ↓ Bone resorption

↑ indicates the acceleration or increase of a given process/substance; ↓ indicates the deceleration or decrease of a given process/substance.

## Data Availability

Not applicable.
